# Impact of Fish Oil and Flaxseed Oil on HIF‐2α, COX‐2 and IFN‐γ Protein Expression: A Case Study in Rats With Kidney Damage Due to Circadian Rhythm Disorder

**DOI:** 10.1002/vms3.70535

**Published:** 2025-07-28

**Authors:** Zeki Erol, Jerina Rugji, Ayşe Aydoğan, Özlem Özmen, Fulya Taşçı

**Affiliations:** ^1^ Department of Food Hygiene and Technology Faculty of Veterinary Medicine Dokuz Eylül University, Kiraz İzmir Turkey; ^2^ Department of Food Science University of Wisconsin‐Madison Madison USA; ^3^ Department of Histology and Embryology Faculty of Veterinary Medicine Necmettin Erbakan University Ereğli Konya Turkey; ^4^ Department of Pathology Faculty of Veterinary Medicine Burdur Mehmet Akif Ersoy University Burdur Turkey; ^5^ Department of Food Hygiene and Technology Burdur Mehmet Akif Ersoy University Burdur Turkey

**Keywords:** circadian rhythm disorder, fish oil, flaxseed oil, kidney damage, signalling protein

## Abstract

**Objectives:**

This study set out to investigate kidney damage by examining the levels of key proteins‐HIF‐2α, COX‐2 and IFN‐γ in rats with disrupted circadian rhythms. It also explored how fish oil and flaxseed oil might influence the intensity of inflammation and its effects on kidney health.

**Methods:**

Circadian rhythm disorder model groups consisted of eight rats each. Except for the control group, other groups (circadian rhythm, flaxseed oil and fish oil groups) were exposed to an adjustable light/dark cycle for 19 days, while the control group was kept on a standard light/dark cycle. In addition to standard food and water ad libitum nutrition for 19 days in four groups, 1000 mg/kg/day flaxseed oil was given to the flaxseed oil group and 400 mg/kg/day fish oil was given to the fish oil group by gavage for 19 days. Histological lesions in tissues were evaluated by Haematoxylin–Eosin staining and using a sequential grading system. For immunohistochemical analysis, IFN‐γ, COX‐2 and HIF‐2 antibodies were used to evaluate the degree of immunohistochemical reactivity of the cells.

**Results:**

Nutrition unequivocally plays a critical role in regulating circadian rhythms, as factors such as meal timing, nutrient composition and gut health directly influence the body's internal clock and overall rhythm stability. Omega‐3 fatty acids are known to influence immune, inflammatory and neurological pathways‐all of which are controlled by the circadian clock. When circadian rhythms are disrupted, key signalling proteins such as HIF‐2α, COX‐2 and IFN‐γ become dysregulated, leading to potential kidney damage. Remarkably, treatments with fish oil and flaxseed oil have been shown to reduce inflammation markers, effectively mitigating kidney damage and offering significant anti‐inflammatory benefits. These results emphasize the aptitude of incorporating fish oil and flaxseed oil into the diet as a powerful strategy to combat kidney damage and slow the pathological effects caused by circadian rhythm disruption.

**Conclusion:**

Circadian rhythm disturbances are associated with various health problems, including kidney damage characterized by microhaemorrhage and inflammatory cell infiltration. In this study, fish oil and flaxseed oil demonstrated protective, anti‐inflammatory effects by reducing levels of HIF‐2α, COX‐2 and IFN‐γ and improving kidney pathology. These effects were evaluated within the scope of an animal model, and the findings provide valuable preliminary data for future human studies.

## Graphical abstract

1. This investigation divulges how disruption in circadian rhythms lead to the disequilibrium of critical signalling proteins like HIF‐2α, COX‐2 and IFN‐γ, contributing to kidney damage and inflammation.

2. Treatment with omega‐3 rich fish oil and flaxseed oil significantly reduced inflammation markers and kidney damage, demonstrating their potential as effective nutritional interventions for combating kidney inflammation.

3. The findings underscore the importance of incorporating fish oil and flaxseed oil into the diet, offering a promising approach to mitigate kidney damage and slow the progression of inflammation caused by disrupted circadian rhythms.

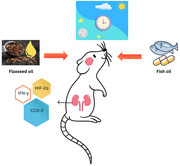

## 1 Introduction

Circadian rhythms (CR), also referred as biological clocks, are internal regulators that organize physiological and behavioural patterns. They enable organisms to adjust to changes in their environment within a 24‐h cycle (Ansu Baidoo and Knutson 2023). The notion ‘circadian rhythms’ derives from the Latin phrase ’circa diem,’ which translates to around the day (Lewis et al. 2020). The circadian system includes a principal clock located in the suprachiasmatic nucleus (SCN) within the hypothalamus, as well as several peripheral clocks situated in various body tissues, including the gastrointestinal tract, liver, pancreas, skeletal muscle and adipose tissue (Öney and Balcı 2021).

The influence of nutrition on CRs is a well‐established concept, with factors such as meal timing, nutrient composition and gut health contributing to maintaining and enhancing these rhythms. Notably, omega‐3 fatty acids are known to impact immune, inflammatory and neurological pathways, all of which are regulated by the circadian clock (Checa‐Ros and D'Marco 2022). A body of research highlights the potential of omega‐3 fatty acids to function as non‐photic zeitgebers, suggesting they may be therapeutically advantageous for treating conditions associated with circadian disruption (Buchhorn et al. 2018; Greco et al. 2014; Wang et al. 2018). Fish oil (FO), as well as various plant seeds like flaxseed, peanuts and soybean oil, are all rich sources of omega‐3 fatty acids (Nordøy 1991). These fatty acids are found in three forms: short‐chain alpha‐linolenic acid (ALA), long‐chain eicosapentaenoic acid (EPA) and docosahexaenoic acid (DHA). ALA is essential and must be obtained through diet, while the body can transform ALA into EPA and DHA, although this process is inefficient (Kocatepe and Turan 2018). Consequently, EPA and DHA should be sourced externally, primarily from fatty fish and flaxseed oil (F), which has the highest ALA content among edible oils (Punia et al. 2019). It is widely recognized that omega‐3 fatty acids influence immune/inflammatory and neurological pathways; these processes are as well regulated by the circadian clock (Checa‐Ros and D'Marco 2022). Existing research indicates that F primarily contains ALA, a short‐chain omega‐3 fatty acid, whereas FO supplies EPA and DHA, which are long‐chain omega‐3 fatty acids. These long‐chain forms are more efficiently absorbed and metabolized by the body and have demonstrated stronger anti‐inflammatory properties (Othman et al. 2008). Furthermore, a separate study observed that although F supplementation raised plasma concentrations of EPA and docosapentaenoic acid (DPA), there was no significant change in plasma DHA levels, suggesting that the conversion of ALA to DHA in the human body is limited (Harper et al. 2006).

Disruption of CRs can lead to dysregulation of key signalling proteins like HIF‐2α, COX‐2 and IFN‐γ, impacting the body's responses to hypoxia, inflammation and immune function, and potentially increasing the risk of chronic diseases. There is evidence of interaction between CRs and hypoxia‐induced signalling, indicating that molecular clocks help the body adapt to changes in nutrient and oxygen levels (Tepebaşı and Calaboğlu 2016; Rankin and Giaccia 2008). Hypoxia‐inducible factors (HIFs) are crucial for oxygen delivery and adaptation to low oxygen levels, with three members identified in human cells: HIF‐1, HIF‐2 and HIF‐3. HIF‐2α is expressed in specific cell types, such as endothelial and kidney fibroblasts, and its activation can reduce inflammation and promote tissue repair (Kapitsinou et al. 2014). COX‐2, typically absent under normal conditions, becomes active in response to various stimuli and plays a key role in inflammation by synthesizing prostaglandins (Kotnik et al. 2005; J. Li et al. 2005). Interferon‐gamma (IFN‐γ) is important for both innate and adaptive immunity, especially against viral and bacterial infections, with high levels associated with inflammation and autoimmune disorders. It is produced by natural killer cells and T cells, reflecting both innate and acquired immune responses (Schroder et al. 2004).

In this work we aimed to i) explore kidney damage by analysing the expressions of HIF‐2α, COX‐2 and IFN‐γ proteins in rats with disrupted CRs, ii) assess the impact of FO and F on the severity of inflammation.

## 2 Materials and Methods

### 2.1 Animals and Ethical Approval

Ethical approval for the study was granted by the Local Ethics Committee for Animal Experiments at Burdur Mehmet Akif Ersoy University (MAKU) (28.08.2024, decision no: 1359). In this study, 32 female Sprague–Dawley rats, aged 8 weeks and weighing an average of 200–300 grams, were assessed. Rats were kept in standard cages for 10 days with a 12:12 light/dark cycle to ensure adaptation. During the adaptation process, the rats were fed standard rat chow and tap water ad libitum. Rats were kept at 21°C–23°C and 55%–60% humidity. Finally, they were randomly divided into four groups with 8 individuals per group. All stages of the study were carried out in the MAKU Faculty of Veterinary Medicine, Pathology Department Laboratory.

### 2.2 Animal Experimental Design

**FIGURE 1:**
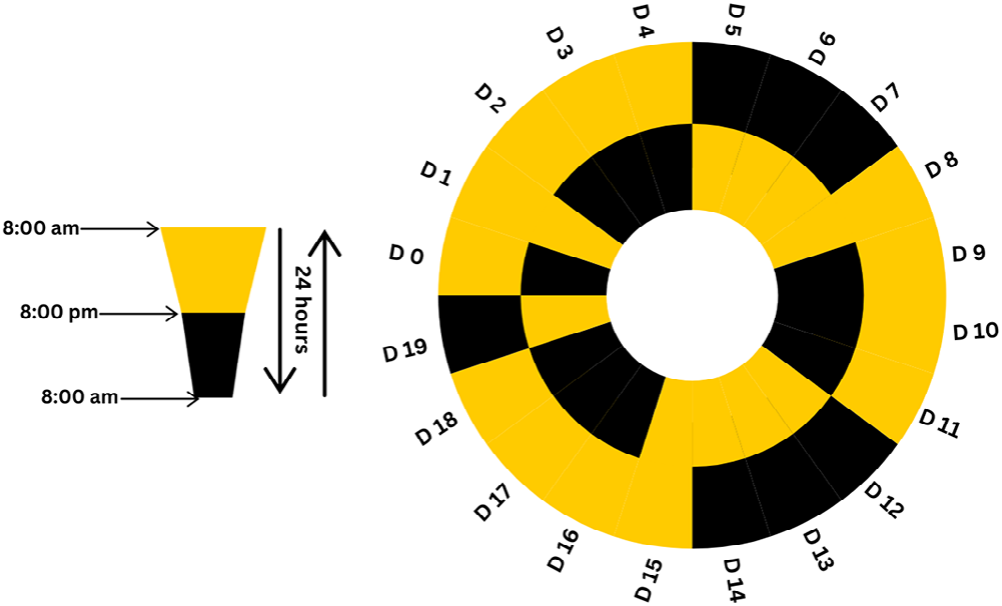
Light/dark hours applied to each group to establish circadian rhythm disorder.

The CR disorder model established in the current investigation was based on the protocol established by Tsai et al. (2005). The research included four distinct groups, each consisting of eight individuals. All groups, except for the control group (C), were housed in specially designed cages with adjustable light/dark cycles to modify their CRs according to the light‐dark patterns. Each group was labelled based on their treatment: the control group (C) were left in the same light/dark cycle, fed with standard chow and water, and considered with normal CR; the CR group was fed standard chow and given tap water ad libitum for 19 days while their CR was disrupted in the special cages; the F group received F (R&D‐Health and Sleep) at a dosage of 1000 mg/kg/day (Abdel Moneim et al. 2011; Görmez 2018) via oral gavage for 19 days, with their CR also disrupted; and the FOoil group was administered FO (Omega 3 Acid Triglycerides, Orzax) at 400 mg/kg/day (Gülcen et al. 2012; Saada et al. 2014) via oral gavage for 19 days, similarly disrupting their CR. In this study, the doses of flaxseed and FO that are considered safe for humans were converted to rodent equivalents using the formula proposed by (Nair and Jacob 2016), which is widely accepted for interspecies dose translation. In addition to this approach, data from previously published studies were also evaluated to determine an appropriate dosing regimen, and the relevant doses were selected accordingly (Nair and Jacob 2016). The protocol presented in Figure 1 was applied to establish CR disorder in the CR, F and FO groups. At the termination of the experimental protocol, the rats were euthanized by cervical dislocation under xylazine (8–10 mg/kg) (Xylazinbio %2, Interhas) and ketamine (80–90 mg/kg) (Ketasol %10, Richter Pharma AG) anaesthesia and their kidney tissues were taken. Kidney tissue samples were fixed in 10% formalin to be used in histopathological and immunohistochemical (IHC) examinations. The fixed tissues were passed through graded alcohol series, methyl benzoate and benzols and blocked in paraffin.

### 2.3 Histopathological Analysis

After being collected during necropsy, kidney samples were visually inspected for any abnormalities and then fixed in a 10% neutral formalin solution. The tissue samples were subsequently embedded in paraffin and processed using automated tissue processing equipment (Leica ASP300S). Once cooled, the paraffin blocks were cut into 5 µm thick sections with a rotary microtome (Leica RM2155). These sections were then stained with Haematoxylin–Eosin (HE) and examined under a light microscope. An ordinal grading system was employed to evaluate histological lesions in the tissues, assessing haemorrhage, hyperaemia, inflammatory cell infiltrations, degenerative changes and necrotic alterations. Tissues were assigned scores ranging from 0 to 3, with mild, moderate and severe affections receiving intermediate values. Table 1 provided an explanation of the grading criteria for the histopathological alterations.

**TABLE 1:** Histopathological grading scores.

Score	Description
0	No lesion
1	Mild hyperaemia, slight haemorrhage, no inflammation and vacuolar degeneration
2	Severe hyperaemia, slight haemorrhage, slight inflammation and slight necrosis
3	Severe hyperaemia, severe haemorrhage, marked inflammation and marked necrosis

### 2.4 Immunohistochemical Examination

Additionally, three sets of sections from each block were transferred to poly‐L‐lysine coated slides and subjected to IHC staining with the following antibodies: IFN‐γ (anti‐interferon gamma antibody [ab7740]), anti‐HIF‐2‐alpha antibody [BL‐95‐1A2]—BSA free (ab272041) and anti‐COX‐2/cyclooxygenase 2 antibody [EPR12012] (ab179800). Following a 60‐min incubation with a 1/100 dilution of the primary antibodies, biotinylated secondary antibodies and a streptavidin‐alkaline phosphatase conjugate were used for immunohistochemistry. The secondary antibody utilized was the EXPOSE Mouse and Rabbit Specific HRP/DAB Detection IHC kit (ab80436), and the chromogen used was diaminobenzidine (DAB) (Abcam, Cambridge, UK). Negative controls were treated with antigen dilution solution instead of primary antibodies. Sections were individually inspected for the IHC analysis of each antibody. The degree of IHC reactivity of the cells was semi quantitatively evaluated using a grading system from (0) to (3) based on the following criteria: (0) *negative staining*, (1) *focal weak staining*, (2) *diffuse weak staining* and (3) *diffuse strong staining*. A total of 10 different areas were assessed in each slice using an objective magnification of 40×. The Database Manual Cell Sens Life Science Imaging Software System was used for morphometric analysis and microphotography (Olympus Co., Tokyo, Japan). Immuno‐positive cell scores were evaluated using the Image J 1.46r program (National Institutes of Health, Bethesda, MD).

### 2.5 Statistical Analysis

Initially, the Shapiro–Wilk method was employed to assess the normality of the data distribution. ANOVA was employed as a means of comparing the groups since the data showed a normal distribution (*p *> 0.05). The histopathology and immunohistochemistry scores among the groups were statistically analysed using one‐way ANOVA with post hoc Duncan test from the SPSS‐22.00 software. A significance level of *p* < 0.05 was applied.

## 3 Results

**FIGURE 2:**
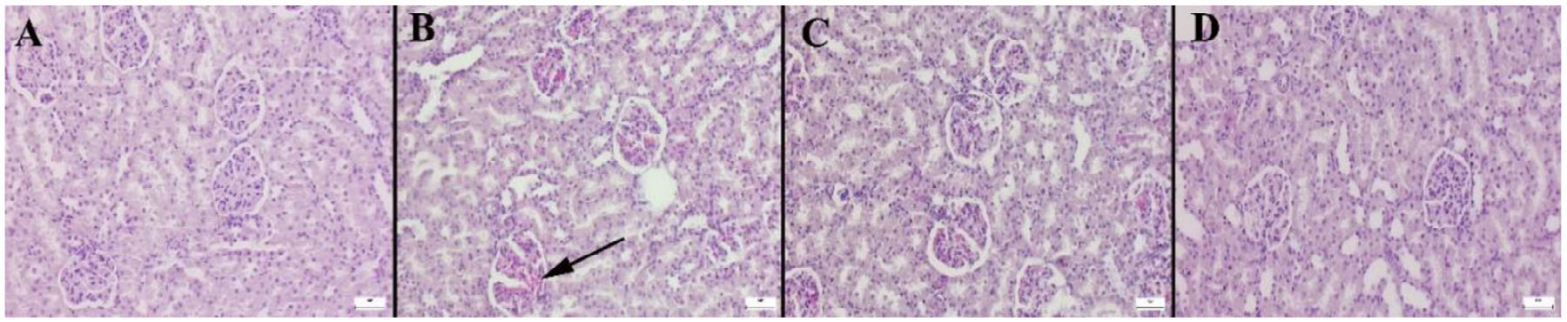
Histopathological appearance of the kidney between the groups. (A) Normal tissue histology in C group. (B) Marked hyperaemia and haemorrhage (arrow) in CR group. (C) No lesions in FO group. (D) Decreased pathological findings in F group. HE, Scale Bars = 50 µm.

**FIGURE 3:**
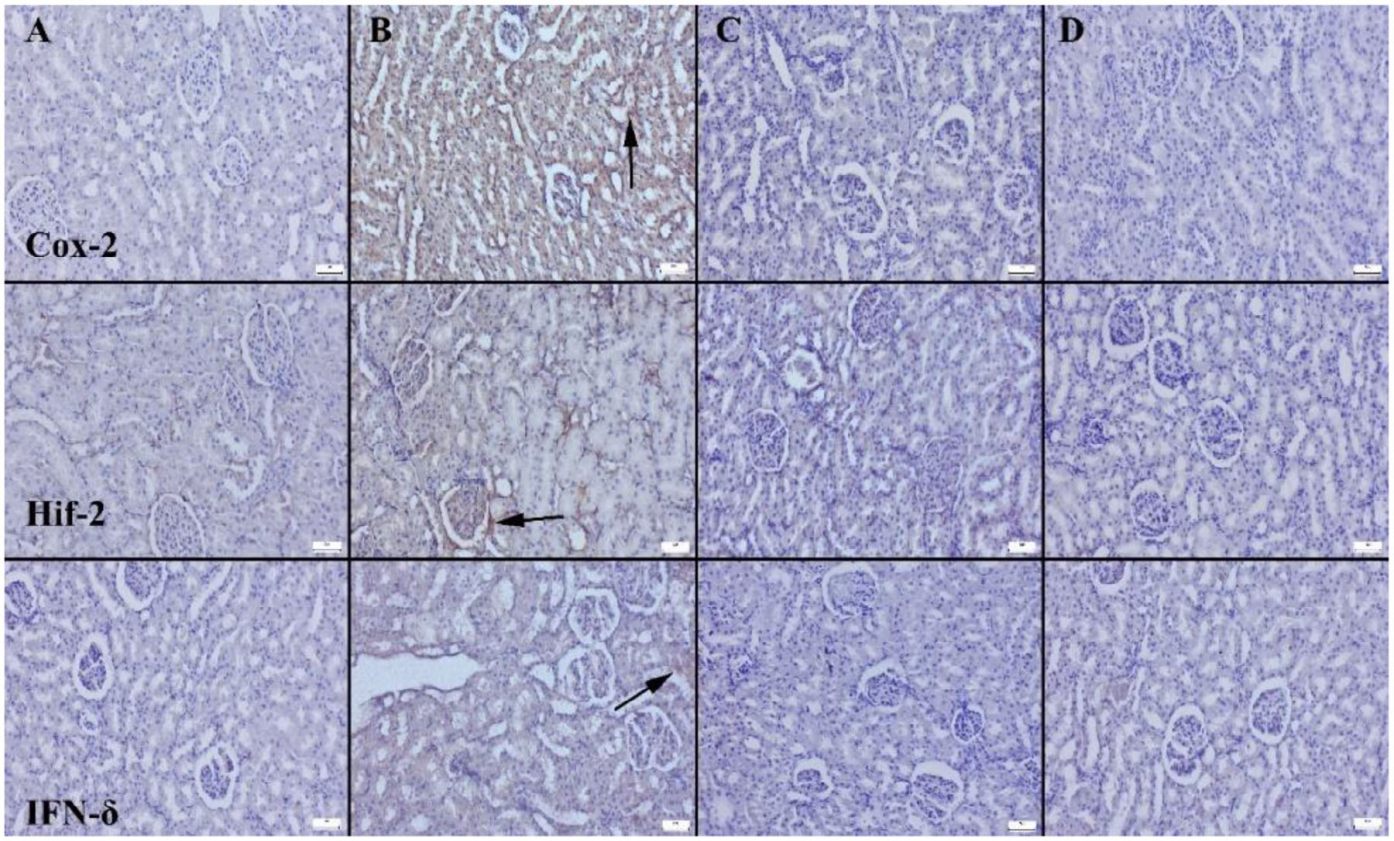
Representative COX‐2, HIF‐2 and IFN‐γ immunohistochemical findings among the groups. (A) Negative expressions in control group. (B) Marked increased in expressions in CR group. (D) Negative expressions in FO group, (C) Almost negative expressions in F group. Streptavidin Biotin Peroxidase Method, Scale Bars = 50 µm.

Histopathological and IHC analysis (IHC) results provide valuable insights regarding the effect of the evaluated compounds on inflammation severity. Histological staining of the collected tissues indicated no evident abnormalities in the kidneys of the individuals belonging to control group (C). Noticeable hyperaemia, microhaemorrhage, and signs of mild to moderate inflammatory cell infiltration were present in the tissues of the individuals belonging to CR group (Figure 2). Pathological findings were significantly improved in the individuals belonging to the groups with disrupted CRs that received treatment with FO and F (Figure 3). Interestingly, results indicated that FO was more potent in reducing these pathological findings compared to F. Table 2 provides the statistical analysis results for the immunohistochemistry scores. Assessment of the signalling proteins HIF‐2α, COX‐2 and IFN‐γ presented a notable increase in expression in the CR group, whereas the C group exhibited few to no immune‐positive cells. IHC staining results showed a significant increase in HIF‐2α expression in the CR group, with minimal immunoreactivity in the control group. However, both the FO and F receiving individuals exhibited a notable decrease in immunoreactivity. Immunoreactivity was present in interstitial, tubular epithelial and endothelial cells across all groups. Moreover, IHC results from the current investigation provided also valuable insights regarding the COX‐2 expression. An essential escalation in COX‐2 expression in the CR group, while negligible immunoreactivity was found in the C group. Immunoreactivity was noted in interstitial cells, tubular epithelial cells and endothelial cells of the kidney. Reduction in COX‐2 expression suggests a decrease in inflammation severity. IHC staining revealed significantly elevated IFN‐γ expression in the CR group, with minimal or absent expression in the C group. Treatment with FO and F resulted in a notable reduction in immunoreactivity. Reactions were primarily observed in interstitial cells, tubular epithelial cells and endothelial cells of the kidney. These findings suggest that the increase in IFN‐γ expression in the CR group and its decrease in treatment groups indicate that FO and F can help prevent or reduce inflammation, potentially slowing down or preventing pathological processes in kidney diseases.

**TABLE 2:** Statistical analysis result of histopathological and immunohistochemical scores between the groups.

Groups	C	CR	FO	*F*	*p* values
Histopathology	0.14 ± 0.14^a^	2.0 ± 0.21^b^	0.28 ± 0.18^a^	0.42 ± 0.20^a^	< 0.001
COX‐2	0.14 ± 0.14^a^	1.28 ± 0.48^b^	0.28 ± 0.18^a^	0.42 ± 0.20^a^	< 0.05
HİF‐3	0.14 ± 0.14^a^	2.14 ± 0.69^b^	0.28 ± 0.18^a^	0.42 ± 0.20^a^	< 0.001
IFN‐γ	0.14 ± 0.14^a^	1.71 ± 0.35^b^	0.28 ± 0.18^a^	0.57 ± 0.20^a^	< 0.001

^a^ The differences between the means of groups carrying different letters between the groups are statistically significant, *p *< 0.001.

^b^ Data expressed mean ± standard deviation (SD). One‐way ANOVA Duncan test. Control group (C) were left in the same light/dark cycle, fed with standard food and water and considered with normal circadian rhythm; the circadian rhythm group (CR) was fed standard rat chow and given tap water ad libitum for 19 days while their circadian rhythm was disrupted in the special cages; the flaxseed oil group (F) received flaxseed oil at a dosage of 1000 mg/kg/day via oral gavage for 19 days, with their circadian rhythm also disrupted; and the Fish Oil group (FO) was administered fish oil at 400 mg/kg/day via oral gavage for 19 days, similarly disrupting their circadian rhythm.

## 4 Discussion

The synchronization of CRs employs a chain of events that consist in the sequencing of internal biological clocks with external signals known as environmental cues or ‘zeitgebers.’ Despite the daily light/dark cycle being the main synchronizing factor, some nutrients, such as food derived omega‐3 fatty acids contribute as non‐photic zeitgebers (Harper et al. 2006). Experimental research has provided important insights into how omega‐3 LC‐PUFAs might help regulate melatonin levels and maintain the integrity of neuronal membranes, both of which are vital for starting and sustaining sleep (Kim et al. 1999; Zhang et al. 1998). Furthermore, human studies examining the integration of omega‐3 LC‐PUFA supplements to the diet have found that a diet rich in omega‐3 LC‐PUFAs (like fatty fish) is linked with earlier sleep onset, longer sleep duration on weekends and better sleep quality (Dashti et al. 2015; Del Brutto et al. 2016; Jansen et al. 2019). Additional research indicates that omega‐3 fatty acids can also decrease proteinuria in patients with chronic glomerular disease and immunoglobulin A (IgA) nephropathy (Behl and Kotwani 2017; Chewcharat et al. 2020). Khan et al. (2012) found that consuming food rich in omega‐3 fatty acids provided protection against nephrotoxicity induced by sodium nitroprusside in rats. Gülcen et al. (2012), reported that omega‐3 fatty acids enhanced the antioxidant defence system in kidney tissue, preventing oxidative damage and providing a protective effect on the kidneys. Previous studies have highlighted the protective role of omega‐3 fatty acids in preventing oxidative damage to the lungs and kidneys caused by various toxic substances, thereby supporting kidney health (Zararsız et al. 2004; Zararsiz et al. 2006). Intake of FO during pregnancy has demonstrated to reduce the overexpression of HIF‐1α (Ghotbeddin et al. 2022). HIFs are key regulators of oxygen balance, influencing development, postnatal physiology and disease mechanisms. HIF‐2α is primarily expressed in the endothelium, kidney, lung, heart and small intestine, with notable expression in endothelial cells and kidney fibroblasts during inflammation (Rosenberger et al. 2002). Moreover, supplementation with FO has been associated with decreased COX‐2 activity (Cugno et al. 2021). The COX‐2 enzyme has a very important role in the inflammatory process and is involved in the production of PGs that play a role in the inflammatory response (Ling et al. 2005). A study examining the effects of a DHA‐rich diet in male Wistar rats found that it led to an increase in serum IFN‐γ, suggesting an immunomodulatory effect (Diau et al. 2024). IFN‐γ is a proinflammatory cytokine essential for both intrinsic and adaptive immunity against viral and bacterial infections, with high levels linked to inflammation and autoimmune diseases (Schoenborn and Wilson 2007).

Flaxseed (*Linum usitatissimum* L.) is a perennial plant recognized for its versatility, largely due to its high levels of omega‐3 polyunsaturated fatty acids (PUFAs), particularly α‐linolenic acid (ALA). It typically contains 35%–45% oil, which comprises 9%–10% saturated fatty acids (palmitic and stearic), about 20% monounsaturated fatty acids (primarily oleic acid), and over 70% ALA. F is well‐known for its health benefits, attributed to its distinctive chemical composition, rendering it one of the richest plant‐based sources of polyunsaturated omega‐3 fatty acids (Al‐Madhagy et al. 2023). Scientific evidence highlights that flaxseed has been shown to decrease pro‐inflammatory cytokines such as IL‐6, IL‐1, TNF‐α and NF‐κB, influencing inflammation and apoptosis processes (M. Li et al. 2011; Pal and Ghosh 2012). Histopathological evaluation indicate that flaxseed reduces necrosis and mitigates inflammatory and oxidative damage in kidney tissue (Al Za'abi et al. 2021). Research has determined that oral administration of F has been associated with improved histopathological outcomes and the inhibition of inflammatory processes and oxidative stress linked to toxicity (Badawy et al. 2015; Makni et al. 2010; Palla et al. 2016; Zhu et al. 2018). These results reinforce our hypothesis that consumption of supplements such as FO and F have a protective effect on kidney damage resulting from CR disorder. The stronger anti‐inflammatory effect of FO compared to F can be explained by the composition and bioavailability of their omega‐3 fatty acids. FO is rich in long‐chain omega‐3 PUFAs, primarily EPA and DHA. These fatty acids are precursors to specialized lipid mediators such as resolvins and protectins, which are known to directly and potently suppress inflammation and actively regulate its resolution. In contrast, F mainly contains ALA, a short‐chain omega‐3 fatty acid that must be enzymatically converted to EPA and DHA in the body. However, this conversion occurs with low efficiency in mammals, particularly in rodents and humans. Consequently, the anti‐inflammatory effects of F may be limited due to the relatively low levels of EPA and DHA generated in vivo (Burdge and Calder 2005). Further comprehensive studies are needed on this subject.

## 5 Conclusion

The influence of CR on overall health is unequivocal, with research indicating that disruptions in CRs are associated to various diseases. This study aimed to address kidney damage by examining the expressions of HIF‐2α, COX‐2 and IFN‐γ proteins in rats with disrupted CRs, as well as to assess the effects of FO and F on inflammation severity. Microhaemorrhage and inflammatory cell infiltration were noted in the kidney tissue of the individuals with disrupted CRs. Obtained data suggests that FO and F offer protection against kidney damage due to their anti‐inflammatory properties. Both FO and F reduced kidney damage and improved pathological findings associated with CR disruptions. Furthermore, the reduction in COX‐2, HIF‐2 and IFN‐γ (inflammatory markers) indicates that the FO and F used in treatment effectively address kidney damage and demonstrate anti‐inflammatory effects by alleviating inflammation caused by various pathological factors. In this study, the effects of CR disruption on renal pathological findings were evaluated, and the efficacy of plant‐based and animal‐based omega‐3 sources was compared. It was found that the animal‐derived FO was more effective than the plant‐derived F in mitigating the renal damage induced by CR disturbance. Substantially, the potential protective effects of FO and F were evaluated within the scope of an animal model used to understand the impact of CR disruptions on the kidneys, and the findings provide valuable preliminary data for future human studies.

## Author Contributions

**Zeki Erol**: conceptualization, methodology, software, data curation, supervision, funding acquisition, resources, writing original draft, writing – review and editing. **Jerina Rugji**: software, writing – review and editing. **Ayşe Aydoğan**: writing – review and editing. **Özlem Özmen**: writing original draft, writing – review and editing. **Fulya Taşçı**: writing – review and editing.

## Ethics Statement

The study was granted unanimous approval by the Burdur Mehmet Akif Ersoy University Animal Experiments Local Ethics Committee, under decision number 1359.

## Conflicts of Interest

The authors declare no conflicts of interest.

## Peer Review

The peer review history for this article is available at https://www.webofscience.com/api/gateway/wos/peer‐review/10.1002/vms3.70535.

## Data Availability

The data that support the findings of this study are available from the corresponding author upon reasonable request.
